# Commentary: Spontaneous expression of mirror self-recognition in monkeys after learning precise visual-proprioceptive association for mirror images

**DOI:** 10.3389/fpsyg.2017.01151

**Published:** 2017-07-11

**Authors:** Xiaoli Liu, Ting Liu, Xiaodan Huang

**Affiliations:** ^1^School of Psychology, Nanjing Normal University Nanjing, China; ^2^College of Life Science and Technology, Jinan University Guangzhou, China

**Keywords:** mirror self-recognition, rhesus monkey, mirror mark test, mirror neurons, tooling use

Self-awareness is believed to be limited in human and great apes (Heschl and Burkart, 2006). Attempting to touch the face mark before a mirror is regarded as the evidence of self-recognition, namely the mirror self-recognition (MSR) test (Anderson and Gallup, 2015). The MSR, as an index of self-recognition, indicates the ability for one separates himself from other people, thoughts and environments (Ma et al., 2015). In Gallop's original experiment, when red odorless dye spots were applied to chimpanzees' faces, chimpanzees would touch face marks as well as do self-directed behaviors before a mirror, for instance, performing exaggerated expressions and exploring invisible body parts like nose, eyes or genitals. Does it mean rhesus monkeys lacking for self-awareness when they could not pass the MSR test consistently? It is questioned by scientists that passing the standard test only can prove the existence of MSR positively, while it's hard to conclude the absence of self-recognition if the animal failed, thus modified precise MSR tests were required to eliminate the limitation (Heschl and Burkart, 2006).

In 2015, a pioneer study employed the visual-somatosensory training to facilitate rhesus monkeys to comprehend that the mirror image was itself (Chang et al., 2015). With high-power laser red spots were projected to face, the rhesus monkey, who sit on the mirror-facing head-fixed chair, was able to touch the laser spots successfully according to the somatosensory feeling, which was further paired with food reward to reinforce the learning process. After 12–38 days' training, the touching rate was up to 95%. The monkey maintained this behavior even in absence of laser induced hot feeling, or food reward. The study was the first success to provide direct evidence that rhesus monkey can pass the MSR after training.

In their newest study, the same group of authors improved the visual-proprioceptive association training to motivate monkey to locate the marked spot spontaneously by the instrumental mirror use. In the training, the monkey was in the head-fixation chair when facing the mirror. A low power laser spot was projected to flat boards, and if the monkey touched the spot precisely via the mirror image, food reward was delivered. After 2–4 weeks' training, successful rate was up to 100%, and persisted without reward. Even the light spot was substituted by colored dye mark, the trained monkey still exhibited mark touch in front of the mirror.

To explore that the trained monkey exactly recognized itself in a mirror instead of simply learning the rule of touching marks, the glass/mirror switching experiment was designed, in which a naïve monkey and a trained monkey were released to a same home cage and separated by glass or a mirror. In the glass period, the social interaction was exhibited between both monkeys, but in the mirror-facing time, touching response was only observed in trained monkeys. Furthermore, trained monkeys behaved more mirror-induced genital-related (GR) and genital-unrelated (UR) behaviors than naïve monkeys when facing the mirror wall. Data collected supported the hypothesis: trained rhesus monkeys are capable to recognize themselves via mirror image.

In the visual-somatosensory training, heat-induced high-power spots were projected to monkeys' face, which is controversial that after training, the mark touching response in monkey is a conditioned behavior not spontaneous MSR.

The improved study perfected training devices to help monkeys precisely learn the association between the face mark position and the image by simply using a mirror. Then, the new study approved the transition from the mirror use to mirror-induced self-recognition performance in the trained monkey and demonstrated the ability to use a mirror and MSR are distinguishing cognitive abilities.

Previous studies suggested that monkey species could not exhibit self-recognition behaviors and failed the MSR test (Roma et al., 2007; Macellini et al., 2010). The new methodology in the present study would therefore inspire more studies to explore self-recognition in primates and even non-primates (Salzen and Cornell, 1968; Berry and Parker, 2016; Deregnaucourt and Bovet, 2016). Otherwise, the new strategy is probable to remedy self-recognition disorder in schizophrenia (SZ) via specific training. The cognitive process of self-recognition is disrupted in schizophrenia, resulting in patients fail to distinguish their own movements and thoughts from those of others (Waters et al., 2012). Although cognitive and neurological deficits happened to in SZ patients, this finding might restore self-recognition ability partially (Gallup et al., 2014).

The underlying mechanism of MSR is unknown. Along with human, chimpanzees are inherent of MSR without training, but monkeys not, which possibly means enhance sensory integration to a certain degree that required for MSR. It's possible that mirror neurons, which are discovered in the monkeys and considered the neural mechanism of imitation behaviors, are the reason of generating self-consciousness in rhesus monkey (Chang et al., 2017). In addition, related studies have found the neural basis of MSR involves frontal regions or the right hemisphere in human. It will be helpful to further elucidate mirror related cortical regions of MSR and neural circuit changes with fMRI after training protocol (Sugiura et al., 2015), for instance, to understand the acquisition and expression of “learned” self-awareness.

There are still some limitations in the present study. Tooling use is an evident indicator of high intelligence in human and high-rank primate and can be transferred within the group (Bayart and Anderson, 1985; Mercader et al., 2007). It will be interesting to see if this “learned” MSR task can be transferred as well. In addition, how early or how late is this learning ability maintained in a monkey's life? When the transition happened in a monkey from using a mirror instrumentally to spontaneous self-directed behaviors in a mirror? Is this due to the involvement of one specific brain region, probably the key region of self-consciousness? Could the monkey own other types of consciousness other than self-awareness? There will be more than one mirror to learn.

## Author contributions

XL and TL completed the first version of the paper and XH presented suggestions to revise the paper. The workload of the three authors was equal.

### Conflict of interest statement

The authors declare that the research was conducted in the absence of any commercial or financial relationships that could be construed as a potential conflict of interest.
